# Venlafaxine, an anti-depressant drug, induces apoptosis in MV3 human melanoma cells through JNK1/2-Nur77 signaling pathway

**DOI:** 10.3389/fphar.2022.1080412

**Published:** 2023-01-04

**Authors:** Ting Niu, Zhiying Wei, Jiao Fu, Shu Chen, Ru Wang, Yuya Wang, Ruihe Zheng

**Affiliations:** ^1^ Central Laboratory, Hainan General Hospital, Hainan Affiliated Hospital of Hainan Medical University, Haikou, China; ^2^ Department of Pharmacy, Hainan General Hospital, Hainan Affiliated Hospital of Hainan Medical University, Haikou, China

**Keywords:** melanoma, Nur77, venlafaxine, drug repurposing, JNK1/2 kinase

## Abstract

**Introduction:** Venlafaxine is one of the most commonly used anti-depressant and antineoplastic drug. Previous studies have predicted venlafaxine as an anti-cancer compound, but the therapeutic effects of venlafaxine in melanoma have not yet been demonstrated. Nur77 is an orphan nuclear receptor that highly expressed in melanoma cells and can interact with Bcl-2 to convert Bcl-2 from an antiapoptotic to a pro-apoptotic protein.

**Method:** We examined the effects of venlafaxine in MV3 cells *in vitro* and MV3 xenograft tumor in nude mice. Western-blot, PCR, TUNEL assay and immunofluorescence were used to reveal the growth of melanoma cells.

**Results:** Here, our data revealed that venlafaxine could reduce the growth, and induce apoptosis of melanoma cells through a Nur77-dependent way. Our results also showed that treatment with venlafaxine (20 mg/kg, i.p.) potently inhibited the growth of melanoma cells in nude mice. Mechanistically, venlafaxine activated JNK1/2 signaling, induced Nur77 expressions and mitochondrial localization, thereby promoting apoptosis of melanoma cells. Knockdown of Nur77 and JNK1/2, or inhibition of JNK1/2 signaling with its inhibitor SP600125 attenuated the anti-cancer effects of venlafaxine.

**Conclusion:** In summary, our results suggested venlafaxine as a potential therapy for melanoma.

## 1 Introduction

Melanoma is the leading cause of skin cancer death worldwide (Cullen et al., 2020). Currently, BRAF inhibitor vemurafenib and MEK inhibitor trametinib are the most common drugs used for the treatment of melanoma (Guo et al., 2021; Chiavarini et al., 2022; Porcelli et al., 2022). However, BRAF-mutated melanomas treated with these compounds almost invariably develop resistance (Siegel et al., 2020). Further research on the molecular mechanism of melanoma and the development of novel therapeutics with high efficiency and low toxicity is highly desired. Nur77 is an orphan nuclear receptor that widely expressed in different types of tumors, including melanoma (Hsu et al., 2004; To et al., 2012). Nur77 plays diverse roles in the regulation of cell proliferation, survival, and apoptosis (Hu et al., 2017; Wu and Chen, 2018; Safe and Karki, 2021). The mitogenic and survival effect of Nur77 may be associated with its transcriptional activity in the nucleus (Zhang, 2007). On the other hand, the pro-apoptotic effect of Nur77 involves its translocation from the nucleus to mitochondria, where it interacts with Bcl-2 and converts Bcl-2 from a survival to a killer of cancer cells (Lin et al., 2004; Kolluri et al., 2008; Zhou et al., 2014; Liu et al., 2017). These complex effects of Nur77 appear to be dependent on its posttranslational modifications (Zhou et al., 2014; Liu et al., 2017). For example, phosphorylation of Nur77 by protein kinase B (AKT) promotes its nuclear shuttling, resulting in the promotion of cancer cell proliferation and invasion, while its phosphorylation by c-Jun N-terminal kinase (JNK) involves the apoptosis in certain cancer cells (Han et al., 2006; Bourhis et al., 2008; Bliss et al., 2012). Thus, targeting Nur77 may offer new strategies to develop effective melanoma therapeutics.

**FIGURE 1:**
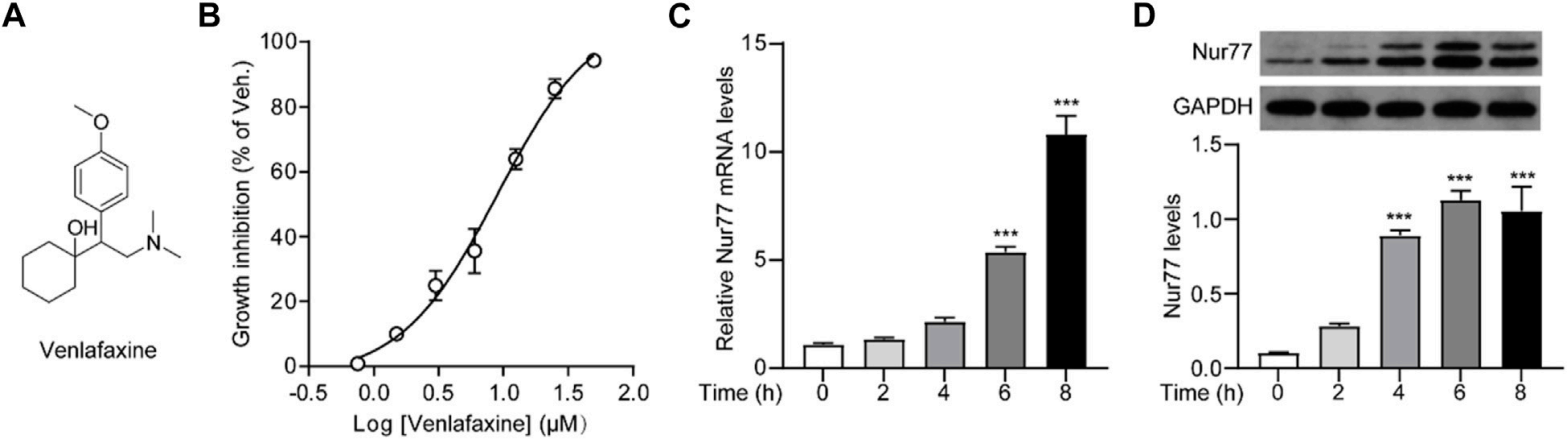
Cellular viability was correlated to Nur77 expression induced by venlafaxine. **(A)** Chemical structure of venlafaxine. **(B)** Dose-dependent inhibition of venlafaxine on the growth inhibition of MV3 cells at 72 h. **(C)** Relative mRNA expressions of Nur77 in MV3 cells were quantified after treatment with venlafaxine (10 μM) for 0–8 h. **(D)** The protein expression of Nur77 in MV3 cells was detected by Western blot after treatment with venlafaxine (10 μM) for 0–8 h. Intensity of the protein bands was quantified and normalized to loading control GAPDH. N = 3, ***, *p* < .001 vs. control (0 h).

To date, many potent Nur77 modulators have been developed (Crean and Murphy, 2021). However, it is extremely challenging to push these compounds towards clinical application. One way to expedite drug development is to discover new uses for approved or investigational drugs. Recently, a study conducted by Bennett et al. revealed that predicted venlafaxine as an anti-cancer compound (Bennett et al., 2022). Venlafaxine is a serotonin and norepinephrine reuptake inhibitor (Figure 1A) and has been used in therapy as an anti-depressant drug since 1993 (Roseboom and Kalin, 2000). Venlafaxine also can attenuate neuropathic pain and vasomotor symptoms in women after cancer (Pinkerton and Santen, 2019; van den Beuken-van Everdingen et al., 2017). Many serotonin reuptake inhibitors, e.g., fluoxetine and desipramine could inhibit melanoma solid tumor growth *in vitro* (Kubera et al., 2009; Grygier et al., 2013). Thus, it is possible that venlafaxine can reduce melanoma cell proliferation. In addition, venlafaxine contains a two-ring group, which is commonly seen in many potent Nur77 modulators, i.g., BI1071 and Triclosan (Ha et al., 2018; Chen et al., 2019). Venlafaxine is likely to act as a Nur77 modulator, exhibiting anti-cancer activities by regulation of Nur77 signaling. So far, venlafaxine has not been explored as a therapeutic approach for melanoma and whether venlafaxine can inhibit the growth of tumor cells in animals is still unknown.

As a proof of concept, we examined the capability of venlafaxine to inhibit the growth of MV3 melanoma cells, and its effects in theNur77 expression. We also studied the molecular mechanisms involved in venlafaxine-induced MV3 cell death. Our results showed that venlafaxine could reduce the growth and induce apoptosis of MV3 cells through the JNK1/2-Nur77 signaling pathway. Our results suggested venlafaxine as a potential therapy for melanoma.

## 2 Materials and methods

### 2.1 Chemicals

All reagents were purchased from Sinopharm (Shanghai, China) unless otherwise indicated. Venlafaxine hydrochloride (Cat. #V129637), SP600125 (Cat. #S125267) and PD98059 (Cat. #P126620) were purchased from Aladdin (Shanghai, China).

### 2.2 Cell culture and treatment

Human melanoma (MV3) cells were purchased from the China center for type culture collection (Cat. #GDC0615). MV3 cells were cultured in DMEM medium supplemented with 10% FBS, 100 units/ml penicillin, and 100 μg/ml streptomycin in humidified 5% CO_2_ atmosphere at 37°C until 80% confluence. MV3 cells were then treated with venlafaxine (0–100 μM), SP600125 (.5 μM) and PD98059 (15 μM) (a MEK/ERK inhibitor that can inhibit MEK activation and subsequent ERK phosphorylation) for 30 min, or human Nur77 siRNA (50 nM), JNK1/2 siRNA (Cell signaling, Cat. #6232S, 50 nM), ERK1/2 siRNA (Cell signaling, Cat. #6560S, 50 nM) and HiPerfect transfection reagent (Qiagen, 301704, United States) for 12 h, followed by incubation at 37°C for 0–72 h (Li et al., 2018). The siRNA sequences were described below:

Control siRNA: 5′-GCGCGCUUUGTAGGAUUCGdTdT-3′

Nur77 siRNA: 5′-CAGUCCAGCCAUGCUCCUCdTdT-3′

### 2.3 Cell viability

Cell viability was measured at 72 h, using the cell counting kit-8 (CCK8) according to the manufacturer’s instructions (Liu J et al., 2022; Qin et al., 2022). MV3 cells were collected and incubated with a CCK-8 reagent (Dojindo, Cat. #CK04, 5 mg/ml, 10 μl) at 37°C for 60 min. The OD values of the reaction mixture were measured at the wavelength of 450 nm. The cell viability was calculated using the following formula: Cell viability (%) = OD (control) − OD (test)/OD (control) − OD (blank).

### 2.4 Apoptosis assay

Apoptosis assay was conducted at 0, 4 and 8 h, using a dead cell apoptosis kit with Annexin V-FITC and propidium iodide (PI) (Thermo, Cat. #V13241) according to the manufacturer’s instructions (Chen et al., 2019; Li et al., 2022). MV3 cells were washed with PBS, resuspended in binding buffer, incubated with Annexin V-FITC and PI for 15 min according to the kit protocol, and analyzed immediately by cytoFLEX Flow Cytometry System (Beckman-Coulter) using FITC and PerCP.

### 2.5 Animals and treatments

The animal experiments were approved by the Animal Care and Use Committee of Hainan Medical University [Approval No. Med-Eth-Re (2022) 736]. Female BALB/c nude mice (20–25 g) were purchased from Shanghai SLAC Laboratory Animal Co., Ltd. Mice were maintained under specific pathogen-free conditions, group-housed in ventilated cages with controlled temperature (25°C ± 1°C) and relative humidity (55% ± 10%). Standard mouse chow and tap water were provided *ad libitum*. The nude mice were anesthetized by intravenous injection of pentobarbital sodium (25 mg/kg), followed by subcutaneous transplantation of MV3 or Nur77 knockout (Nur77 KO) MV3 melanoma cells in the right posterior axillary line (Liu Y. X et al., 2022). The dose of venlafaxine for the mice was based on the preliminary experiments and the references (Zhang et al., 2019; Madrigal-Bujaidar et al., 2021). Mice were treated with venlafaxine (20 mg/kg, i.p.) or its .1% DMSO-containing saline vehicle once daily after tumor size grew up to 50–100 mm^3^. Body weight and tumor volume were measured every 3 days. Mice were sacrificed by CO_2_ inhalation after 15-day drug treatment and the tumors were stripped for various assessments (Chen et al., 2019). Nur77 knockout (KO) MV3 cells were generated by CRISPR/Cas9 system using a previously reported method (Chen et al., 2019). The gRNA targeting sequence of Nur77 is 5′-ACC​TTC​ATG​GAC​GGC​TAC​AC-3′. Protein lysates were prepared from tumors with previously reported method (Chen et al., 2015). Tumors were homogenized in cold 1× RIPA lysis buffer and centrifuged at 15,000 × g for 15 min at 4°C. The supernatants were collected and the protein concentration of sample was measured by Pierce BCA protein assay kit (Thermo, Cat. #23225). Ultimately, all samples were normalized to the same total protein concentration of 2 mg/ml. Frozen tumor tissues were thawed and then homogenated in Trizol reagent. Total RNA was extracted with chloroform, isopropanol, and 75% ethanol. The RNA concentration was measured with a spectrophotometer (Beckman Coulter, United States).

### 2.6 Western blot

Western blots were performed using the standard sodium dodecyl sulfate (SDS)-PAGE polyacrylamide gel electrophoresis method (Li et al., 2021a). The protein of cell or tumor lysates was prepared and measured by Pierce BCA protein assay kit (Thermo, Cat. #23225) (Xie et al., 2022a). The total protein (50 μg) was separated by 10% SDS-PAGE gels and transferred to a nitrocellulose membrane (Amersham Biosciences, Shanghai, China). Membranes were blocked in 5% (w/v) nonfat milk for 1 h at room temperature, washed with saline buffer (containing .05% tween-20) and then incubated at 4°C overnight with the primary antibody: Nur77 (Cell signaling, Cat. #3960S, dilution 1:500), cleaved caspase-3 (Cell signaling, Cat. #9661S, dilution 1:300), PARP (Santa Cruz, Cat. #sc-8001, dilution 1:500), p38 (Novus, Cat. #AF8691, dilution 1:500), p-p38 (Santa Cruz, Cat. #sc-166182, dilution 1:300), JNK1/2 (Santa Cruz, Cat. #sc-137019, dilution 1:800), p-JNK1/2 (R&D, Cat. #AF1205, dilution 1:400), c-Jun (Novus, Cat. #MAB8930, dilution 1:500), p-c-Jun (Cell signaling, Cat. #3270S, dilution 1:300), ERK1/2 (Cell signaling, Cat. #68303SF, dilution 1:600), p-ERK1/2 (Cell signaling, Cat. #9101S, dilution 1:300), GAPDH (Santa Cruz, Cat. #sc-47724, dilution 1:1000). The membranes were then incubated for 1 h at room temperature with horseradish peroxidase (HRP)-linked anti-rabbit IgG antibody (Santa Cruz, Cat. #sc-2004, dilution 1:5000) and detected with an electrochemiluminescence plus kit (Amersham Biosciences).

### 2.7 Real-time polymerase chain reaction (RT-PCR)

Total RNAs were extracted by Trizol (Invitrogen) and complemental DNA was synthesized using RevertAid First‐Strand cDNA Synthesis Kits (Fermentas). RT-PCR was performed using SYBR Green dye and the Roche LightCycler^®^ 480 II system following the manufacturer’s instructions on a 7300 real-time PCR system (Applied Biosystems) using respective primers (Hu et al., 2021):

Nur77: 5′-ACC​CAC​TTC​TCC​ACA​CCT​TG-3′ (forward), 5′- ACTTGGCGTTTTTCT GCACT-3′ (reverse).

β-Actin: 5′-AGA​GCT​ACG​AGC​TGC​CTG​AC-3’ (forward), 5′-AGCACTGTGTTG GCGTACAG-3′ (reverse).

Expression data were normalized to β-Actin mRNA expression.

### 2.8 Histological analysis

Tumor tissues were excised, sectioned and fixed in 10% (w/v) formalin for 24 h, followed by embedding in paraffin. The specimen was embedded in paraffin and cut into 5 μm sections for further assessments (Xie et al., 2022b).

### 2.9 Immunofluorescence

In vitro–cultured MV3 cells were fixed with 4% paraformaldehyde, permeabilized with .1% Triton X-100 in PBS for 20 min, and then blocked with goat serum in .3 M glycine in PBS at 25°C for 1 h. Sections were then incubated at 4°C overnight with the primary antibody: Nur77 (Cell signaling, Cat. #3960S, dilution 1:500) and Bcl2 (Abcam, Cat. #ab692, dilution 1:500). Sections were rinsed with .1 M PBS and exposed to donkey secondary antibodies conjugated with Alexa Fluor 488 or 647 (Abcam, dilution 1:1,000) at room temperature for 2 h. After an additional rinse, cells were then counterstained with 4′, 6-diamidino-2-phenylindole (DAPI) for nuclear labelling (Wang et al., 2022). Fluorescence images were captured with a confocal microscope.

The paraffin-embedded tumor sections were incubated with the following primary antibodies at 4°C overnight: Nur77 (Cell signaling, Cat. #3960S, dilution 1:500), cleaved caspase-3 (Cell signaling, Cat. #9661S, dilution 1:300), Ki-67 (Abcam, Cat. #ab15580, dilution 1:800). After incubation, sections were rinsed with .1 M PBS and exposed to goat secondary antibodies conjugated with Alexa Fluor 488 or 647 (Abcam, dilution 1:1,000) at room temperature for 1 h (Li et al., 2021b). After an additional rinse, sections were then counterstained with DAPI for nuclear labelling. Fluorescence images were captured with a confocal microscope.

Reactive oxygen species (ROS) were stained with cellular ROS assay kit (deep red) (Abcam, Cat. #ab186029) according to the manufacturer’s instructions. Terminal deoxynucleotidyl transferase-mediated dUTP nick end labeling (TUNEL) assays were carried out according to the manufacturer’s instructions (Promega, Cat. #G7131). The number of apoptotic cells was counted by Image J.

### 2.10 Statistical analysis

Data are presented as means ± SEM. Analyses were performed with GraphPad Prism 9.0.5. Three or more different groups were analyzed by one-way ANOVA with Dunnett’s *post hoc* multiple comparison tests. *p* < .05 was considered statistically significant.

## 3 Results

### 3.1 Cellular viability was correlated to Nur77 expression induced by venlafaxine

We first investigated whether venlafaxine could regulate the cellular viability of MV3 cells. As shown in Figure 1B, venlafaxine showed a great inhibition on the growth of MV3 cells with an LD_50_ = 9.01 ± .97 μM. To investigate whether the cytotoxicity effect of venlafaxine was associated with its induction of Nur77 expression, we then examined the effect of venlafaxine on Nur77 expression in MV3 cells. Based on the LD_50_ data of venlafaxine, MV3 cells were treated with venlafaxine at a concentration of 10 μM. qRT-PCR (Figure 1C) and western blot (Figure 1D) analyses showed that the mRNA and protein levels of Nur77 were low in MV3 cells, but were persistently elevated after venlafaxine treatment. Together, these results suggested that venlafaxine reduced the cell proliferation and introduced Nur77 expression in MV3 cells.

### 3.2 Venlafaxine-induced apoptosis in MV3 cells

**FIGURE 2:**
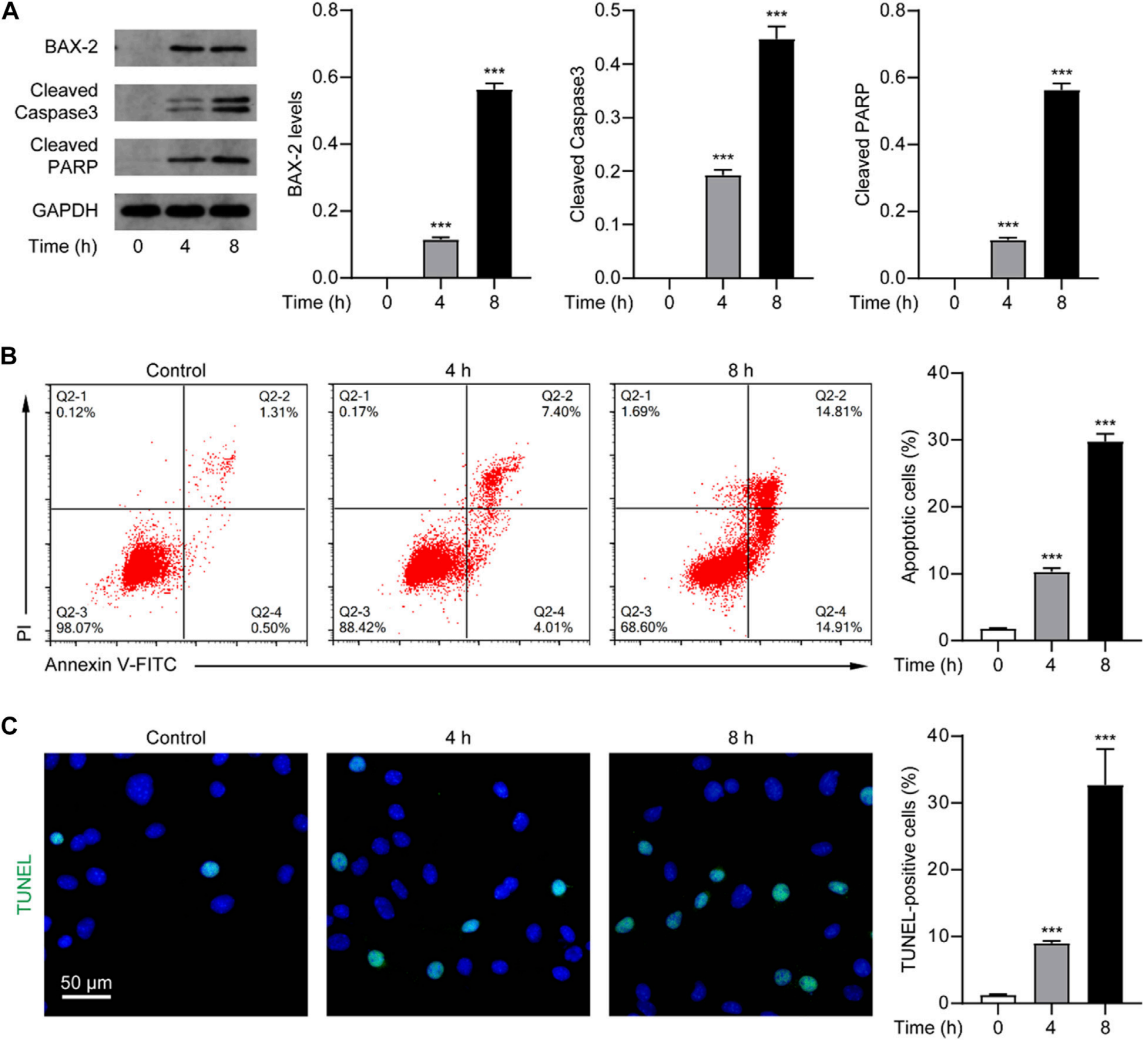
Venlafaxine induced apoptosis in MV3 cells. **(A)** The protein expression of BAX-2, cleaved caspase 3 and cleaved PARP in MV3 cells was detected by Western blot after treatment with venlafaxine (10 μM) for 0–8 h. Intensity of the protein bands was quantified and normalized to loading control GAPDH. **(B)** Apoptosis assays in MV3 cells treated with venlafaxine (10 μM) for 0–8 h were conducted by flow cytometry. **(C)** The apoptotic cells were detected by TUNEL assay in MV3 cells treated with venlafaxine (10 μM) for 0–8 h. N = 3, ***, *p* < .001 vs. control (0 h).

Given that the death effect of Nur77 is associated with its induction of apoptosis, which has been observed in other studies (Zhang, 2007), we speculate that venlafaxine may exert an anti-proliferative effect through activating the Nur77-dependent apoptotic pathway. As a proof of concept, we examined the expression of BAX-2, cleaved caspase 3 and cleaved PARP, the indicators of apoptosis, in MV3 cells treated with venlafaxine (10 μM). Western-blot analysis showed that venlafaxine treatment persistently induced caspase 3 and PARP cleavage and BAX-2 expression in MV3 cells (Figure 2A). We further assessed the effect of venlafaxine on cell death using flow cytometry-based Annexin V/Propidium iodide (PI) apoptosis assay. As shown in Figure 2B, approximately 30% of MV3 cells were apoptotic when treated with venlafaxine for 8 h, whereas only about 2% of cells were apoptotic in vehicle control group. Moreover, TUNEL assay revealed extensive apoptosis in venlafaxine-treated MV3 cells (Figure 2C). Together, these results revealed that venlafaxine induces apoptosis in MV3 cells.

### 3.3 Venlafaxine induced Nur77 mitochondrial targeting and ROS production in MV3 cells

**FIGURE 3:**
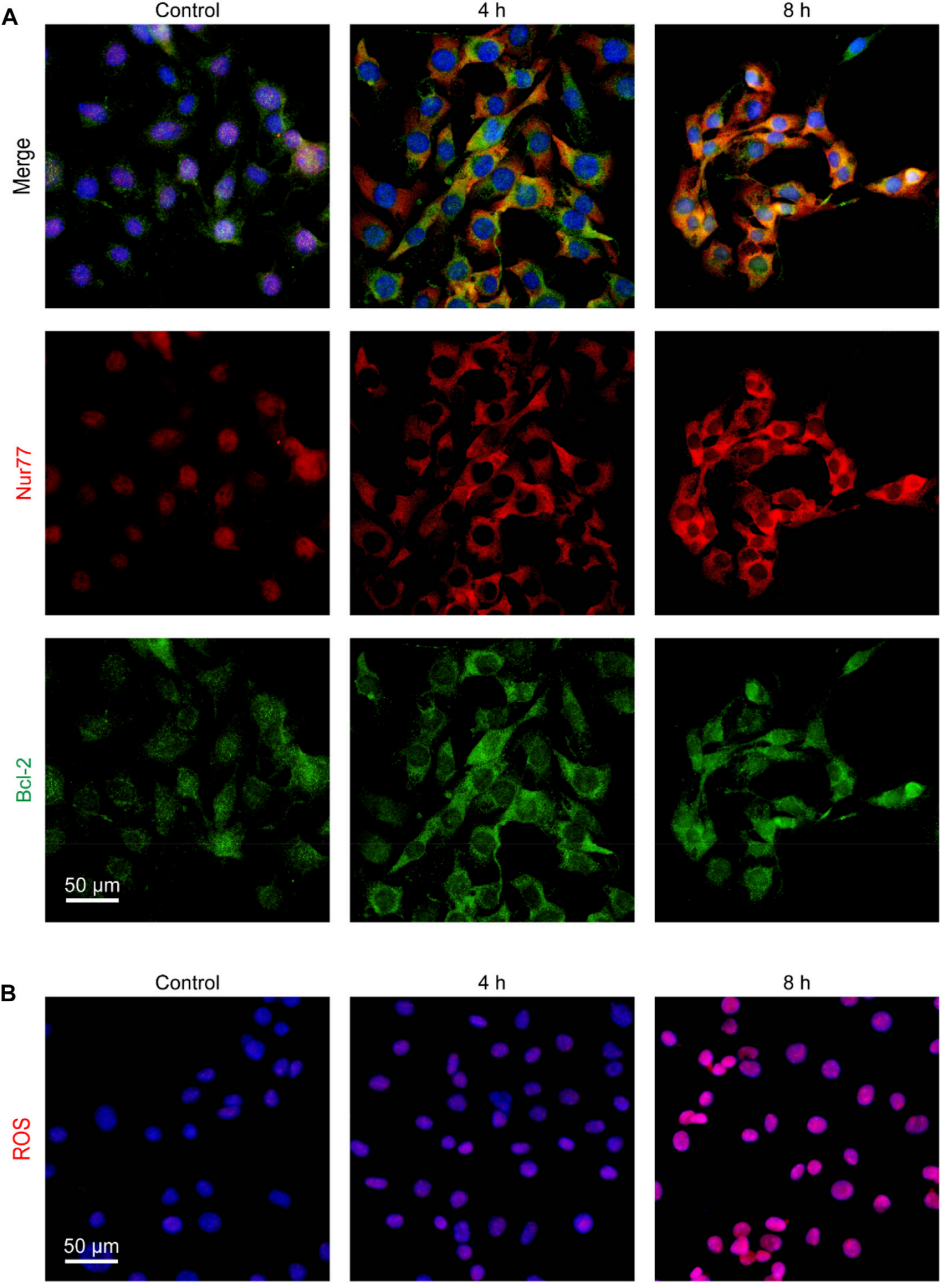
Venlafaxine induced Nur77 mitochondrial targeting and ROS production in MV3 cells. **(A)** MV3 cells treated with venlafaxine (10 μM) for 0–8 h were immunostained with Bcl-2 and Nur77 antibodies and visualized by confocal microscopy. **(B)** MV3 cells treated with the cellular ROS assay kit and visualized by confocal microscopy.

Nur77 can induce apoptosis by translocating to mitochondria where it binds to Bcl-2 to trigger cytochrome c release and ROS production (Lin et al., 2004; Chen et al., 2019). Thus, we further examined whether venlafaxine could induce Nur77 translocation from the nucleus to mitochondria. Immunofluorescence assay showed that Nur77 was mainly localized in the nucleus of MV3 cells, while treatment of venlafaxine promoted the mitochondrial translocation of this protein (Figure 3A). Furthermore, following Nur77 mitochondrial translocation, the production of ROS in MV3 cells was also enhanced by venlafaxine (Figure 3B). Together, these results revealed that venlafaxine induced Nur77 mitochondrial targeting and ROS production in MV3 cells.

### 3.4 Nur77 expression was necessary for venlafaxine-induced apoptosis

**FIGURE 4:**
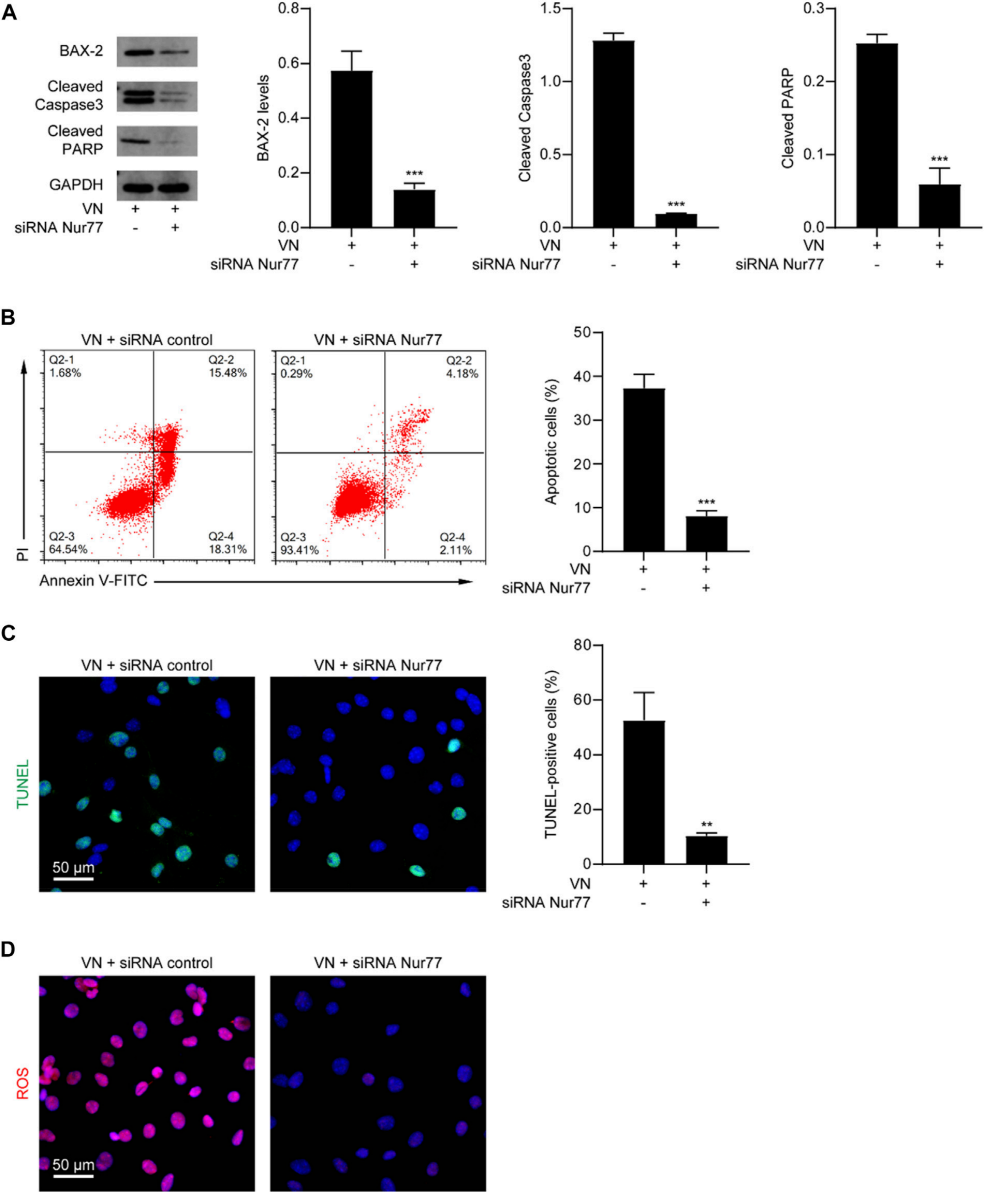
Nur77 expression was necessary for venlafaxine-induced apoptosis. MV3 cells were transfected with siRNA control or Nur77 siRNA, following by treatment with venlafaxine (10 μM) for 0–8 h. **(A)** The protein expression of BAX-2, caspase 3 and cleaved PARP in MV3 cells was detected by western blot. Inte cleaved nsity of the protein bands was quantified and normalized to loading control GAPDH. **(B)** Apoptosis assays in MV3 cells were conducted by flow cytometry. **(C)** The apoptotic cells were detected by TUNEL assay. **(D)** MV3 cells treated with the cellular ROS assay kit and visualized by confocal microscopy. N = 3, **, *p* < .01, ***, *p* < .001 vs. control.

We next determined whether venlafaxine-induced apoptosis was Nur77-dependent. MV3 cells were transfected with Nur77 siRNA and subjected to venlafaxine treatment for 8 h. We found that Nur77 knockdown decreased venlafaxine-induced expressions of BAX-2, cleaved caspase 3 and cleaved PARP were in MV3 cells (Figure 4A). Furthermore, venlafaxine-induced apoptosis was rescued by transfecting with Nur77 siRNA, but not control siRNA (Figures 4B, C). Additionally, venlafaxine induced lower levels of ROS in Nur77-knockdown cells than control MV3 cells (Figure 4D). Together, these data indicated that Nur77 expression was necessary for venlafaxine-induced apoptosis.

### 3.5 Activation of JNK1/2 signaling was necessary for venlafaxine-induced cell death effects

**FIGURE 5:**
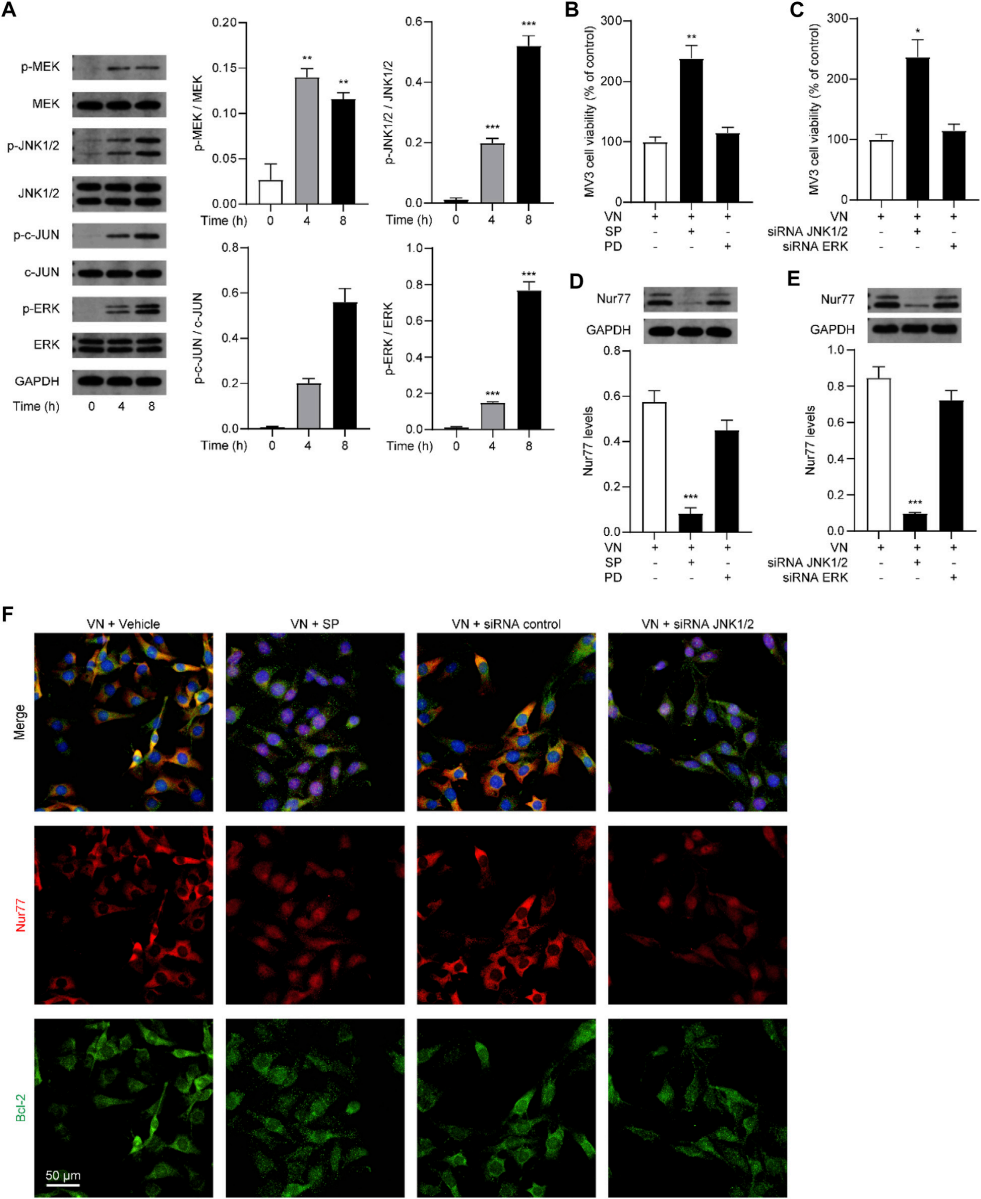
Activation of ERK1/2 signaling was necessary for venlafaxine-induced apoptosis. **(A)** Representative western-blot bands and quantification of the MAPK signal molecules abundances in MV3 cells treated with venlafaxine (10 μM) for 0–8 h **(B)** MV3 cells were treated with vehicle, JNK1/2 inhibitor SP600125 and MERK/ERK inhibitor PD98059 for 72 h. Cell viability were measured by CCK-8 kits at 72 h **(C)** MV3 cells were transfected with siRNA control, JNK1/2 siRNA, and ERK siRNA for 72 h. Cell viability were measured by CCK-8 kits at 72 h **(D,E)** Representative western-blot bands and quantification of Nur77 abundances in MV3 cells treated with venlafaxine (10 μM) for 8 h, or transfected with siRNA control, JNK1/2 siRNA, and ERK siRNA for 20 h **(F)** MV3 cells were immunostained with Bcl-2 and Nur77 antibodies and visualized by confocal microscopy. N = 3, *, *p* < .05, **, < .01, ***, *p* < .001 vs. control.

Given that MAPKs play an important role in the regulation of melanoma cell survival and Nur77 post-translational modifications (Liu et al., 2017; Huo et al., 2020; Hu et al., 2021), we further studied whether venlafaxine affects Nur77 expression through MAPKs signaling. MV3 cells expressed relatively low levels of phosphorylated MEK (p-MEK), p-JNK1/2, p-c-Jun, and p-ERK. After treatment with venlafaxine, the levels of p-MEK, p-JNK1/2, p-c-Jun, and p-ERK were increased in MV3 cells (Figure 5A). To further study the roles of MAPK signaling in venlafaxine-induced apoptosis, MV3 cells were co-treated with venlafaxine and different inhibitors of MAPK kinases, including MEK/ERK signaling inhibitor PD98059 and JNK1/2 inhibitor SP600125. CCK8 assay showed that inhibition of JNK1/2 activity by SP600125 impaired venlafaxine-induced cell death effects, while PD98059 had no such effects (Figure 5B). Moreover, knockdown of JNK1/2 but not ERK increased cell viability in venlafaxine-treated MV3 cells (Figure 5C). These data demonstrated that JNK1/2 signaling is involved in venlafaxine-induced cell death effects. Next, we examined whether venlafaxine affects Nur77 expression in MV3 cells through JNK1/2 signaling. As shown in Figures 5D, E, both genetic inactivation and pharmacological administration of JNK1/2 inhibitor SP600125 significantly suppressed venlafaxine-induced Nur77 expression, indicating that venlafaxine increases Nur77 expression in MV3 cells through activation of JNK1/2 signaling. Furthermore, blockade of JNK1/2 signaling also suppressed mitochondrial translocation of Nur77 (Figure 5F). Taken together, these data suggested that venlafaxine induced MV3 cells death through activation of JNK1/2 signaling and induction of Nur77 expression.

### 3.6 Anti-cancer efficacy of venlafaxine in mice

**FIGURE 6:**
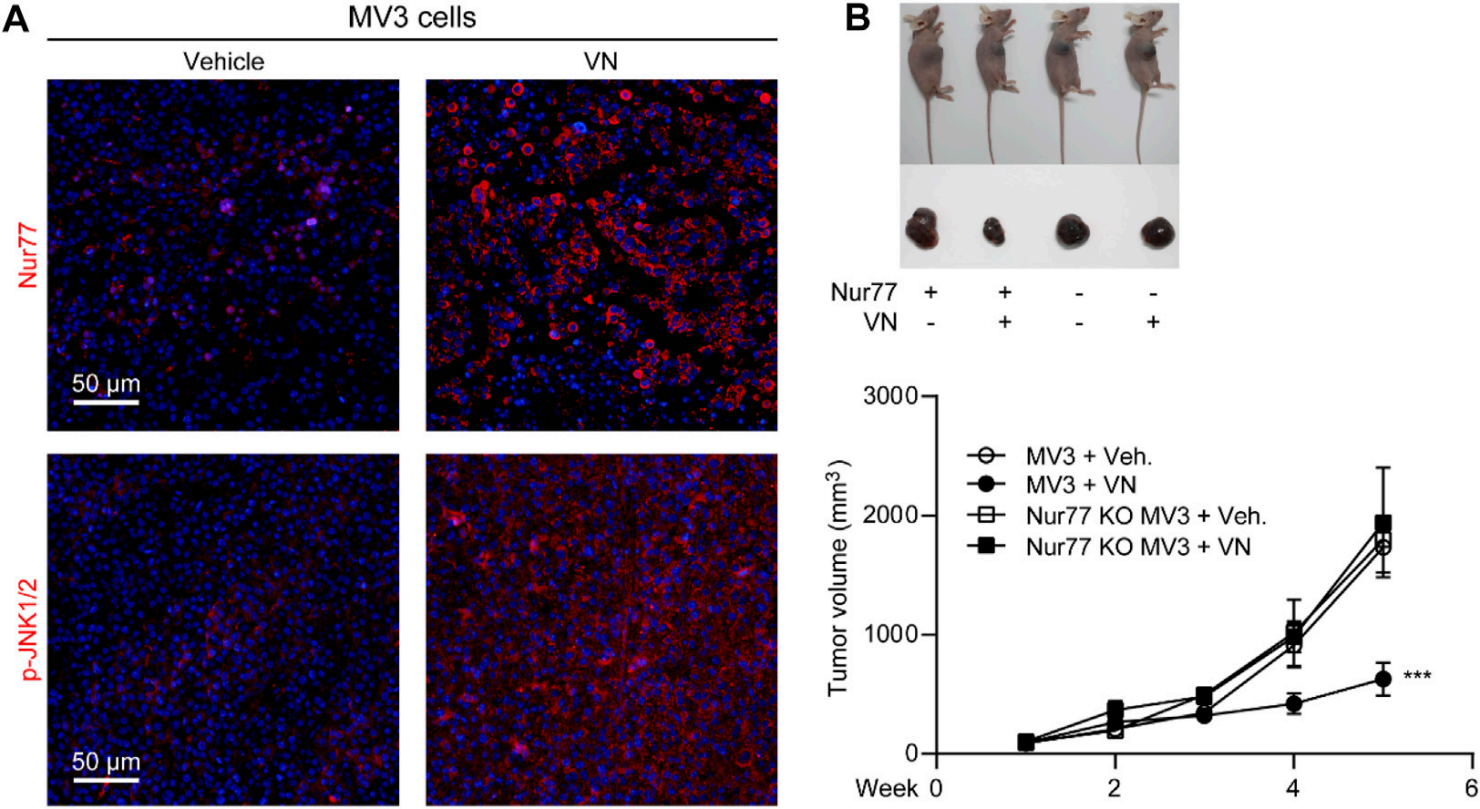
Anti-cancer efficacy of venlafaxine in mice. BALB/c nude mice bearing MV3 or Nur77 KO MV3 xenograft tumors were treated with venlafaxine (20 mg/kg, i.p.) or its vehicle once daily from week 1 to week 5 after implantation of MV3 or Nur77 KO MV3 cells. **(A)** Tumor tissues were immunostained with Nur77 and JNK1/2 antibodies and visualized by confocal microscopy. **(B)** Venlafaxine reduced cancer cell growth in nude mice through Nur77. One of five similar experiments is shown.

Encouraged by the *in vitro* activity of venlafaxine, we further investigated whether the induction of Nur77 by venlafaxine contribute to its growth inhibitory effect *in vivo*. BALB/c nude mice were inoculated subcutaneously with MV3 cells, and were treated with venlafaxine (20 mg/kg, i.p., once daily for 15 days) when the average tumor size grew up to 50–100 mm^3^. Consistent with the *in vitro* results, treatment with venlafaxine for 15 days promoted expressions of Nur77 and activation of JNK1/2 in tumor tissues (Figure 6A). Furthermore, at the 15 days after implantation, the average tumor volume of the vehicle control group was 1,732 mm^3^; and that of venlafaxine treatment group was 630 mm^3^, with the inhibition ratio of tumor growth was 64% (Figure 6B). These results demonstrated that venlafaxine induced Nur77 expression and inhibit the growth of MV3 xenograft tumor *in vivo*.

**FIGURE 7:**
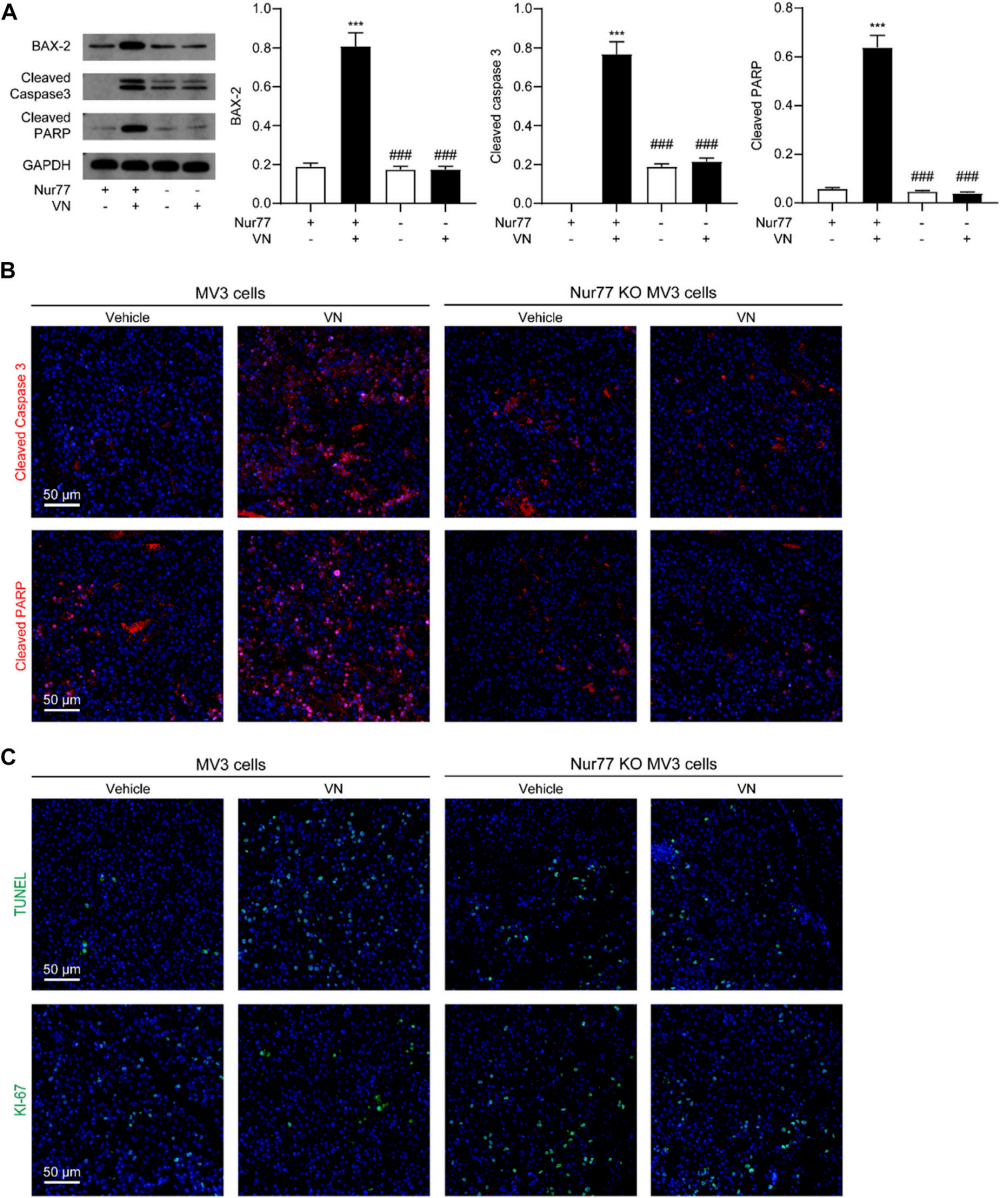
Venlafaxine-induced apoptosis *in vivo*. BALB/c nude mice bearing WT MV3 or Nur77 KO MV3 xenograft tumors were treated with venlafaxine (20 mg/kg, i.p.) or its vehicle once daily from week 1 to week 5 after implantation of MV3 or Nur77 KO MV3 cells. **(A)** Representative western-blot bands and quantification of BAX-2, cleaved caspase 3 and cleaved PARP abundances in tumor tissues. N = 5, *, *p* < .05, **, < .01, ***, *p* < .001 vs. MV3 vehicle control. ^###^, *p* < .001 vs. MV3 venlafaxine control. **(B)** Tumor tissues were immunostained with cleaved caspase 3 and cleaved PARP antibodies and visualized by confocal microscopy. **(C)** Tumor tissues were immunostained with TUNEL assay kits and antibodies and Ki-67 visualized by confocal microscopy. One of five similar experiments is shown.

To study whether venlafaxine inhibit the growth of MV3 xenograft tumor associated with its induction of apoptosis. We examined the expression of BAX-2, cleaved caspase 3 and cleaved PARP in tumor tissues. Western-blot assay and immunostaining showed that venlafaxine treatment significantly increased the level of these protein (Figures 7A, B). Additionally, immunostaining assay demonstrated that venlafaxine enhanced apoptotic cell death, as assessed by TUNEL staining, and suppressed cell proliferation, as assessed by Ki-67 immunostaining, in tumor tissues (Figure 7C). When combined, these results suggested that venlafaxine potently inhibited the growth of melanoma cells in animals through its apoptotic cell death effects.

Furthermore, we also studied the role of Nur77 in the anti-cancer effects of venlafaxine *in vivo*. We used the CRISPR/Cas9 technology to generate Nur77 knockout (KO) MV3 cells, and subcutaneously injected Nur77 KO MV3 melanoma cells into the BALB/c nude mice. At the 15 days after implantation, we found that the average tumor volume of the Nur77 KO MV3 group was similar to that of MV3 group, indicating Nur77 is not required for the growth of MV3 xenograft tumor. Furthermore, treatment with venlafaxine could not inhibit the growth of Nur77 KO MV3 xenograft tumor (Figures 6B, C), as well as the expression of BAX-2, cleaved caspase 3 and cleaved PARP in tumor tissues (Figures 7A, B). Moreover, treatment with venlafaxine had no effects on the numbers of TUNEL-positive and Ki-67-positive cells (Figure 7C). Taken together, these results suggested that venlafaxine suppresses the growth of melanoma cells through Nur77-dependent apoptotic pathway.

## 4 Discussion

**FIGURE 8:**
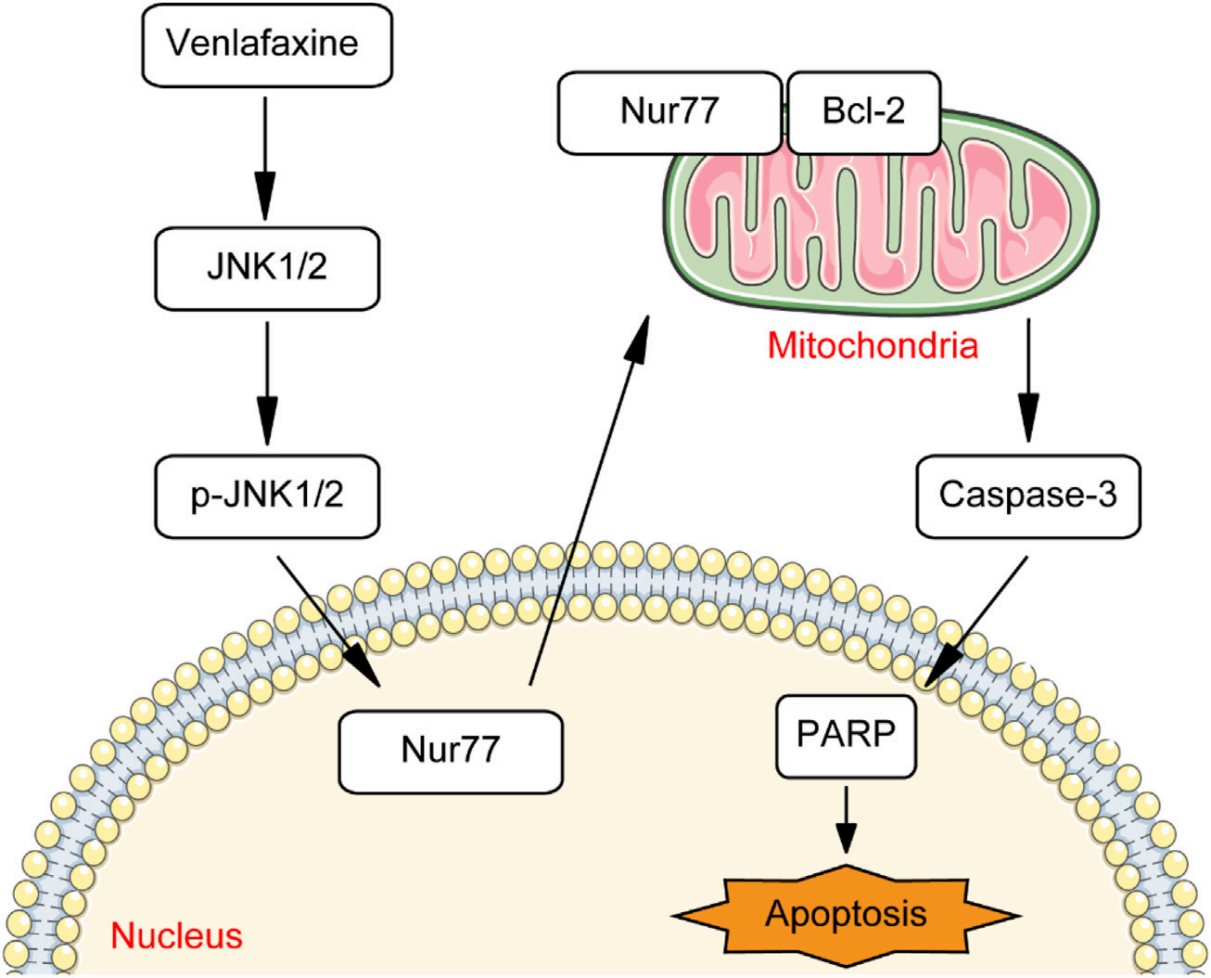
Schematic summary of venlafaxine mediated signaling pathway.

Melanoma is one of the most aggressive and dangerous form of skin cancer (Ahmed et al., 2020). Although immunotherapies and targeted therapies are highly effective in ameliorating melanoma, their clinical use is hindered by the drug resistance (Ahmed et al., 2020). Therefore, novel approaches to melanoma treatment are still highly desired to reduce the mortality rate of patient with melanoma. One way to rapidly develop therapeutic agents for melanoma is drug repurposing, defined as the re-application of known drugs to target new indication. Some of the classic examples of successful repurposing are minoxidil and gabapentin. Minoxidil, originally an anti-hypertensive agent, is now commonly used to promote hair re-growth (Goren and Naccarato, 2018). Gabapentin, originally used as anti-epileptics, is now used to treat neuropathic pain (Moore and Gaines, 2019). Therefore, we switch our attention from newly synthesized anti-cancer compound to approved drugs to rapidly develop therapeutic agents for melanoma. As a proof of concept, we demonstrated that anti-depressant drug venlafaxine could inhibit the growth of MV3 melanoma cells through introduction of Nur77 expression. Mechanistically, venlafaxine activates JNK1/2 signaling pathway, thus trigger expression and mitochondrial localization of Nur77. Follow mitochondrial translocation, Nur77 binds to Bcl-2 and converts Bcl-2 from a survival to a killer of cancer cells, thereby inhibiting the growth and induce apoptosis of MV3 melanoma cells (Figure 8).

A significant finding presented here is that venlafaxine can induce apoptosis of MV3 human melanoma cells through a Nur77-dependent pathway. Nur77 is an orphan nuclear receptor. It often translocate to mitochondria and binds to Bcl-2 in response to different death signals, leading to a conformation change in Bcl-2 and conversion of Bcl-2 from performing an anti-apoptotic role to a pro-apoptotic role (Lin et al., 2004; Kolluri et al., 2008). Many anti-cancer drugs, including vinblastine, vincristine, taxol, and cisplatin also induce expression of Nur77 (Deacon et al., 2003; Achkar et al., 2018). Herein, we found that venlafaxine treatment could increase Nurr77 expression and interaction with Bcl-2 (Figures 1D, 3A), resulting in ROS production (Figure 3B), and apoptosis (Figure 2A). Interestingly, the mRNA expression of Nur77 after 4 h incubation is non-significant, although there is significant protein expression at the same time point (Figures 1C, D). The reason for the discrepancies between mRNA expressions and protein expression is not clear. There may be serval reasons attributed to these results. It has been reported that Nur77 can bind to Bcl-2 after phosphorylation by JNK1/2. It is possible that the certain posttranslational modifications of Nur77 may increase the binding affinity of Bur77 to its antibody, thus a significant band of Nur77 was also observed at 4 h. Furthermore, we cannot exclude the possibility that the degradation of Nur77 was reduced in MV3 cells after 4 h incubation with venlafaxine. Future studies need to be carried out to confirm the precise reason for this result. Furthermore, the Nur77 subgroup of nuclear hormone receptors subfamily has been implicated in the pathophysiology of the central nervous system, including manic depression, Parkinson’s disease, schizophrenia, and Alzheimer’s disease (Liu et al., 2021). Venlafaxine also exhibits pharmacological actions in these diseases (Mazeh et al., 2004; Mokhber et al., 2014; El-Saiy et al., 2022; Zhuo et al., 2022). It is possible that venlafaxine produces therapeutic effects in these diseases through introduction of Nur77 expression.

A number of studies suggested that MAPKs, including JNK1/2 and ERK and their related upstream or downstream signal molecules such as MEK can regulate Nur77 expression (Anjum et al., 2022). Our results showed that venlafaxine could activated the JNK/c-Jun and the MERK/ERK pathway. Both pathways are documented in the literature. For example, c-Jun is a transcription factor that positively modulated Nur77 mitochondrial localization and activation by directly binding to the Nur77 DNA promoter regions (Han et al., 2006; Ming et al., 2018). The transcriptional activity of c-Jun is associated with its abundance and posttranslational modification (Han et al., 2006; Ming et al., 2018). Ha et. al. (2018) have reported that inhibition of c-Jun phosphorylation by miR-6321/Map3k1 reduced Nur77 protein expression, while activation of the JNK/c-Jun pathway by anisomycin increased Nur77 levels (Han et al., 2006). In addition, the MERK/ERK pathway plays a critical role in inducing Nur77 expression. GnRH promoted Nur77 expression in alpha T3-1 cells through ERK signaling (Bliss et al., 2012; Oliveira et al., 2021; Liu Y. X et al., 2022). Scalarane sesterterpenoid 12-deacetyl-12-epi-scalaradial induces HeLa cells apoptosis through ERK-mediated expression and phosphorylation of Nur77 (Zhou et al., 2020). Furthermore, malayoside, a cardenolide glycoside extracted from Antiaris toxicaria Lesch, also induced Nur77 expression, phosphorylation, and human non-small lung cancer cells apoptosis through ERK signaling (Hu et al., 2021). Our results showed that venlafaxine-induced Nur77 expression and cell apoptosis can be blocked by treatment with JNK1/2 inhibitor SP600125, but not MERK/ERK inhibitor PD98059, suggesting that venlafaxine may induced MV3 cell apoptosis through the JNK/c-Jun signaling.

## 5 Conclusion

In conclusion, our results demonstrate that venlafaxine could reduce the growth and induce apoptosis of MV3 cells through the JNK1/2-Nur77 signaling pathway. Our results also identified venlafaxine as a promising therapy for melanoma.

## Data Availability

The raw data supporting the conclusion of this article will be made available by the authors, without undue reservation.
